# The relationship between inotrope exposure, six-hour postoperative physiological variables, hospital mortality and renal dysfunction in patients undergoing cardiac surgery

**DOI:** 10.1186/cc10302

**Published:** 2011-07-07

**Authors:** Jason Shahin, Benoit deVarennes, Chun Wing Tse, Dan-Alexandru Amarica, Sandra Dial

**Affiliations:** 1 Division of Critical Care, McGill University Health Centre, 687 Pine Avenue West, Montreal, QC, H3A 1A1, Canada; 2 Division of Cardiac Surgery, McGill University Health Centre, 687 Pine Avenue West, Montreal, QC, H3A 1A1, Canada; 3 McGill University Faculty of Medicine, 845 Sherbrooke Street West, Montreal, QC, H3A 2T5, Canada; 4 Respiratory Epidemiology and Clinical Research Unit, Montreal Chest Institute, McGill University Health Centre, 3650 St-Urbain, Montreal, QC, H2X 2P4, Canada; 5 Department of Critical Care, SMBD-Jewish General Hospital, McGill University, 845 Sherbrooke Street West, Montreal, QC, H3A 2T5, Canada

## Abstract

**Introduction:**

Acute haemodynamic complications are common after cardiac surgery and optimal perioperative use of inotropic agents, typically guided by haemodynamic variables, remains controversial. The aim of this study was to examine the relationship of inotrope use to hospital mortality and renal dysfunction.

**Material and methods:**

A retrospective cohort study of 1,326 cardiac surgery patients was carried out at two university-affiliated ICUs. Multivariable logistic regression analysis and propensity matching were performed to evaluate whether inotrope exposure was independently associated with mortality and renal dysfunction.

**Results:**

Patients exposed to inotropes had a higher mortality rate than those not exposed. After adjusting for differences in Parsonnet score, left ventricular ejection fraction, perioperative intraaortic balloon pump use, bypass time, reoperation and cardiac index, inotrope exposure appeared to be independently associated with increased hospital mortality (adjusted odds ratio (OR) 2.3, 95% confidence interval (95% CI) 1.2 to 4.5) and renal dysfunction (adjusted OR 2.7, 95% CI 1.5 to 4.6). A propensity score-matched analysis similarly demonstrated that death and renal dysfunction were significantly more likely to occur in patients exposed to inotropes (*P *= 0.01).

**Conclusions:**

Postoperative inotrope exposure was independently associated with worse outcomes in this cohort study. Further research is needed to better elucidate the appropriate use of inotropes in cardiac surgery.

## Introduction

Low cardiac output syndrome is a common complication in patients undergoing cardiac surgery [1]. The aetiology is multifactorial and is believed to be related to a combination of myocardial ischaemia, reperfusion injury, cardioplegia-induced myocardial dysfunction and preexisting cardiac disease [2]. The administration of fluids and inotropes and the use of ventricular assist devices are common therapies in the management of low cardiac output syndrome. Physiological variables such as cardiac index, mixed venous oxygen saturation, oxygen delivery and lactate levels, obtained either from a cardiac output monitoring device or by measuring oxygen consumption and delivery, are often used to guide therapy. Inotropic agents are frequently used and titrated to achieve certain target levels of these physiological variables [3].

However, there is no consensus regarding low cardiac output syndrome in terms of both the physiological parameters that define it and the interventions used to treat it [2]. The use of inotropes has been described to be both centre- and physician-dependent and as being administered to as few as 5% or to as many as 100% of patients undergoing elective coronary bypass surgery [4-6]. Although inotropes have been demonstrated to improve haemodynamics and measured physiological variables [2], they may be a source of increased mortality and morbidity as they can increase cardiac arrhythmias and ischaemia [7]. Very few randomised, controlled trials comparing the different agents used and their effects on clinical outcomes have been performed in patients who undergo cardiac surgery [2]. In a recent observational study, the receipt of dobutamine perioperatively was associated with increased mortality [8]. In addition, investigators in randomised, controlled trials of inotropes in patients with heart failure and left ventricular dysfunction have reported increased side effects and increased mortality [9].

While the use of inotropes as part of a protocol to target physiological parameters within the first six hours after cardiac surgery has been shown to improve outcomes in patients with sepsis [10], it is unclear whether their use in the postoperative cardiac care setting is favourable with respect to morbidity and mortality. This study was undertaken to evaluate the relationship of inotrope use to morbidity and mortality in a cohort of consecutive patients undergoing cardiac surgery.

## Materials and methods

### Setting and study population

This study was conducted at two adult tertiary care university-affiliated hospitals. Data were collected retrospectively between 1 January 2005 and 31 December 2005 by trained reviewers using standardised data collection sheets. Consecutive patients who had undergone coronary artery bypass graft (CABG) surgery, valve replacement or repair or combined CABG and valvular or aortic procedures were included in the study. Patients who had undergone a heart transplant, pulmonary thromboendarterectomy or placement of a ventricular assistance device were excluded.

All patients had been admitted postoperatively to the ICU. A Swan-Ganz catheter had been used perioperatively at both hospitals to guide patient resuscitation. Serum lactate and mixed venous oxygen saturation levels had been measured in all patients at one site, and selected patients at the other site. To avoid bias, only the data from the patients treated at the hospital with routinely measured serum lactate and mixed venous oxygen saturation were used for the analysis of those variables.

All research was conducted in keeping with the principles outlined in the Declaration of Helsinki. The research ethics committee of the McGill University Health Centre Research Institute approved the study. The hospital's ethics committee waived the need for informed consent as the data were collected retrospectively.

### Exposure, covariates and end points

The primary drug exposure studied was postoperative inotrope use. Epinephrine, milrinone and dobutamine were the main inotropes used in the study centres. The definition of 'postoperative inotrope use' varied for each medication. Milrinone exposure was defined as administration of any dose for any length of time in the ICU. Dobutamine exposure was defined as delivery of any dose as long as it was administered for at least three hours in the ICU. Epinephrine exposure was defined as a minimum duration of three hours in the ICU if the dose was < 5 μg/minute or any duration if doses ≥ 5 μg/minute were used. Consistent with the definitions used by other authors [2], norepinephrine and vasopressin were not considered inotropes.

The primary study outcomes were hospital mortality and the occurrence of postoperative renal dysfunction. Renal dysfunction was defined by an increase in creatinine ≥ 200% from baseline in the first five postoperative days or new renal replacement therapy at any point during hospitalisation. These definitions are consistent with stages 2 and 3 of the Acute Kidney Injury Network classification system [11]. Secondary outcomes were ICU length of stay and hospital length of stay.

Data were collected regarding patient age, sex, Parsonnet score, medical history, procedure-related variables and six-hour postoperative physiological variables. Medical histories were abstracted from patient records. Conditions considered were any prior cardiac surgery, hypertension, diabetes, atrial fibrillation, preoperative hospitalisation for heart failure, preoperative renal dysfunction, preoperative dialysis, preoperative left ventricular ejection fraction and left ventricular dysfunction. Preoperative renal dysfunction was defined by a preoperative creatinine level ≥ 150 μmol/L. Left ventricular dysfunction was defined as left ventricular ejection fraction < 30%. Procedure-related variables consisted of cardiac procedures, perioperative intraaortic balloon pump use, reoperation, emergency operation, mean bypass time and severe postoperative bleeding. Cardiac procedures were separated into a CABG-only group and a group who had undergone other procedures, defined as (1) valve repair or replacement or (2) combined CABG and valve repair or replacement. An emergency operation was defined as a cardiac procedure occurring within 24 hours of acute coronary syndrome or immediately after a percutaneous intervention. Reoperation was defined as the need for reoperation 48 hours after the initial cardiac procedure. Severe postoperative bleeding was defined as blood loss > 2.5 L within 24 hours after the initial cardiac procedure. The six-hour postoperative physiological variables consisted of mean arterial pressure, cardiac index, mixed venous oxygen saturation and serum lactate. These tests were performed in the ICU six hours after the operation.

### Data analysis

Preoperative variables, intraoperative variables and outcomes in patients exposed or unexposed to inotropes were compared using Student's *t*-test and the Wilcoxon rank-sum test for continuous variables and a χ^2 ^test for categorical variables. Multivariable logistic regression was performed to test the association between inotrope exposure and outcomes after adjusting for possible confounding variables. A forward stepwise procedure was first used to assess inotrope exposure in the model. Variables were kept in the model if they were believed to be clinically important or if they altered the association between inotrope exposure and outcome by ≥ 10%. Covariates included in the model were Parsonnet score, left ventricular dysfunction, perioperative intraaortic balloon pump use, bypass time, reoperation, postoperative bleeding, preoperative renal dysfunction, treatment hospital, aprotinin use and cardiac index < 2.2 [3]. Variables included in the Parsonnet score were not included in the model, other than a low left ventricular ejection fraction, as we thought this might strongly influence decision making with respect to inotrope use, and its weighting in the Parsonnet score might not reflect this. Colinearity between left ventricular ejection fraction and Parsonnet score was excluded as the variance inflation factor was < 2.5.

A second analytic method, propensity score matching, was performed to evaluate the association between inotrope exposure and outcomes. A one-to-many greedy five-to-one digit technique was performed to match one control in the no agent group (control group) by one case (inotrope-exposed). A 'greedy five-to-one digit match' means that the cases were first matched to controls on five digits of the propensity score. For those that did not match, cases were then matched to controls on four digits of the propensity score. This continued down to a one-digit match on propensity scores for those that remained unmatched. If a one-digit match was not possible, the case remained unmatched and was not included in the matched case control analysis. Matching variables included Parsonnet score, low left ventricular ejection fraction, age, sex, bypass time, procedure type, CABG only or other procedures, and perioperative intraaortic balloon pump use. In this matched sample, baseline characteristics and outcomes were compared between inotrope exposed and unexposed groups by performing paired *t*-tests for continuous variables and McNemar's test for categorical values.

Because of previous literature correlating patient outcomes with physiological goals [10] and recommendations that certain levels of physiologic variables be targeted [3], we examined the relationship between measured six-hour physiologic variables and outcomes. Specifically, oxygen delivery, cardiac index, serum lactate and mixed venous oxygen saturation were examined. The six-hour postoperative physiological variables were categorised with cutoffs based on recommendations in the literature [3,10], with the exception of oxygen delivery, which was based on the median value derived from the data. We examined the relationship between inotrope exposure and outcomes after stratifying by the six-hour physiological variables. All data processing and analyses were performed using SAS version 9.2 software (SAS Institute, Cary, NC, USA).

## Results

**Figure 1:**
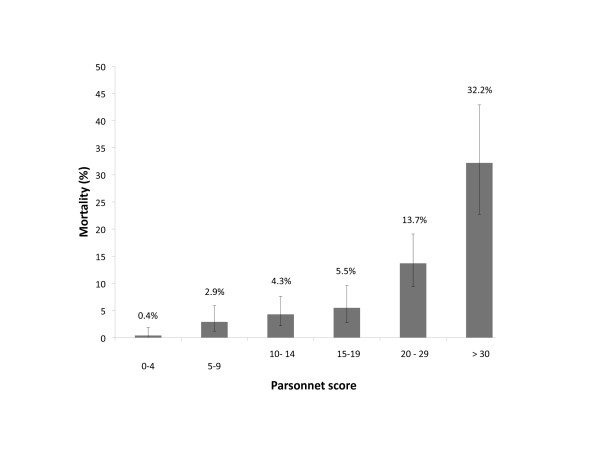
**Hospital mortality by Parsonnet score in a cohort of 1,326 cardiac surgery patients**. Error bars indicate 95% confidence intervals (95% CIs).

In total, 1,326 patients were initially included in the study. Their mean age was 66 years, with 10% of the cohort being older than 80 years of age and more than two-thirds being male. The majority of the procedures were CABG operations, and the mean Parsonnet score (± SD) was 13.4 ± 10.6 (Table 1). Over 97% of the patients had Swan-Ganz catheters inserted. Fifty percent of patients were exposed to inotropes intraoperatively, and forty-two percent were exposed postoperatively. The hospital mortality rate was 7.8%, and renal dysfunction occurred in 8.3% of the patients (Table 2). As shown in Figure 1, hospital mortality increased progressively with Parsonnet scores > 20.

**Table 1:** Baseline characteristics for total cohort and patient subgroups^a^

Characteristics	Total cohort (*N *= 1,326)	Inotrope-unexposed (*n *= 783)	Inotrope-exposed (*n *= 531)
Demographics			
Mean age (± SD)	66.1 (11.0)	64.6 (10.7)	68.3 (11.0)
Age ≥ 80 years, *n *(%)	123 (9.3)	51 (6.5)	72 (13.6)
Females, *n *(%)	404 (30.5)	188 (24.0)	207 (39.0)
Mean Parsonnet score (± SD)	13.4 (10.6)	10.5 (8.3)	17.2 (11.7)
Medical history			
Prior cardiac surgery, *n *(%)	78 (5.9)	26 (3.3)	48 (9.0)
Hypertension, *n *(%)	904 (68.2)	534 (68.2)	362 (68.2)
Diabetes, *n *(%)	417 (31.5)	220 (28.1)	193 (36.4)
Atrial fibrillation, *n *(%)	135 (10.2)	53 (6.8)	80 (15.1)
Preoperative CHF, *n *(%)	179 (13.5)	79 (10.1)	99 (18.6)
Preoperative renal dysfunction, *n *(%)	94 (7.1)	31 (4.0)	61 (11.5)
Preoperative dialysis, *n *(%)	21 (1.6)	9 (0.7)	12 (2.3)
Mean LVEF (± SD)	49.7 (11.8)	53.3 (11.8)	44.4 (15.8)
LVEF < 30%, *n *(%)	241 (18.2)	74 (9.5)	162 (30.5)
Procedure-related variables			
CABG only, *n *(%)	912 (68.8)	601 (76.8)	303 (57.1)
Other procedure, *n *(%)	414 (31.2)	182 (23.2)	228 (42.9)
Perioperative IABP, *n *(%)	132 (10.0)	30 (3.8)	96 (18.1)
Received inotropes intraoperative, *n *(%)	666 (50.7)	219 (28.0)	447 (84.2)
Emergency operation, *n *(%)	132 (10.0)	58 (7.4)	74 (13.9)
Reoperation, *n *(%)	78 (5.9)	20 (2.6)	35 (6.6)
Mean bypass time, minutes (± SD)	101.2 (45.7)	86.8 (32.8)	120 (52)
Severe postoperative bleeding, *n *(%)	50 (3.8)	15 (1.1)	35 (6.6)

^a^CHF, congestive heart failure; LVEF, left ventricular ejection fraction; CABG, coronary artery bypass graft; IABP, intraaortic balloon pump; SD, standard deviation.

**Table 2:** Postoperative variables and outcomes^a^

Variables and outcomes	Total cohort (*N *= 1,314)	Inotrope-unexposed (*n *= 783)	Inotrope-exposed (*n *= 531)
Postoperative inotrope and vasopressor use, *n *(%)			
Norepinephrine	674 (51.3)	279 (35.6)	395 (74.4)
Vasopressin	73 (5.6)	5 (0.6)	68 (12.8)
Epinephrine	308 (23.29)	0	308 (58.0)
Dobutamine	75 (5.7)	0	75 (14.1)
Milrinone	332 (25.3)	0	332 (62.5)
Six-hour postoperative physiological variables			
Mean arterial pressure, mmHg (± SD)	75.9 (10.0)	76.7 (10.0)	74.5 (9.7)
Mean oxygen delivery, mL/minute/m^2^ (± SD)	353 (101)	363 (100)	341 (98)
Mean cardiac index, L/min (± SD)	2.9 (0.7)	2.9 (0.7)	2.8 (0.7)
Mixed venous oxygen saturation, *n *(%)	70.7 (9.0)	71.3 (8.2)	70.1 (9.3)
Mean serum lactate, μmol/L (± SD)	3.2 (2.7)	2.1 (1.6)	4.1 (3.0)
Outcomes			
Died, *n *(%)	103 (7.8)	15 (1.9)	76 (14.3)
Renal dysfunction, *n *(%)	105 (8.3)	25(3.2)	87 (16.8)
Median ICU length of stay, days (IQR)	1.1(1.8)	1.0 (0.9,1.6)	2.1 (1.0,4.7)
Median hospital length of stay, days (IQR)	8 (6)	7.0 (5.0,9.0)	10.0 (6.0,18.0)

^a^IQR, interquartile range; SD, standard deviation.

Twelve patients who died within six hours of admission to the ICU were excluded from further analysis, leaving a total of 1,314 patients finally included in the study.

Patients exposed to inotropes differed from those unexposed in that they were older; had higher Parsonnet scores, more comorbid illnesses and lower preoperative left ventricular ejection fractions; were more likely to have undergone valvular or combined procedures; and had longer bypass times. However, at six hours after ICU admission, aside from serum lactate, which was higher in the group of patients exposed to inotropes, postoperative physiological parameters were very similar. The hospital mortality rate was seven times higher in the group of patients exposed to inotropes compared to those not exposed. Similarly, the rate of severe renal failure was significantly higher in the patients exposed to inotropes (Table 2).

**Figure 2:**
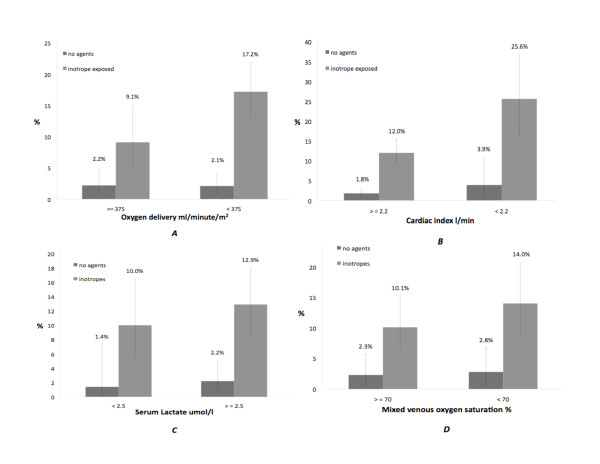
**Hospital mortality stratified by (A) oxygen delivery, (B) cardiac index, (C) serum lactate and (D) mixed venous oxygen saturation**. Two groups of patients are represented (inotrope-exposed and inotrope-unexposed). The adjusted odds ratios for the association between inotrope exposure and mortality were 8.5 (95% CI 4.8 to 15.0) after adjusting for oxygen delivery and 7.7 (95% CI 4.4 to 13.7) after adjusting for cardiac index. The adjusted odds ratios for the association between inotropes exposure and mortality were 5.7 (95% CI 2.4 to 13.5) after adjusting for serum lactate and 5.3 (95% CI 2.4 to 11.4) after adjusting for mixed venous oxygen saturation. Error bars indicate 95% CI. Data from only one hospital were used for analyses of serum lactate and mixed venous oxygen saturation, resulting in wider 95% CIs.

**Figure 3:**
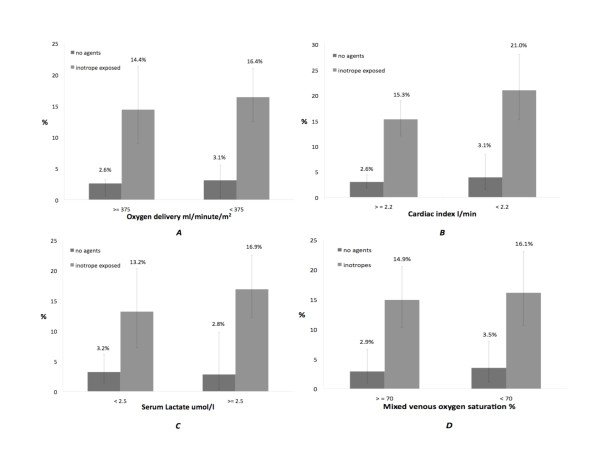
**Renal dysfunction stratified by (A) oxygen delivery, (B) cardiac index, (C) serum lactate and (D) mixed venous oxygen saturation**. Two groups of patients are represented (inotrope-exposed and inotrope-unexposed). The adjusted odds ratios for the association between inotrope exposure and renal dysfunction were 6.0 (95% CI 3.8 to 9.5) after adjusting for oxygen delivery and 5.9 (95% CI 3.7 to 9.6) after adjusting for cardiac index. The adjusted odds ratios for the association between inotrope exposure and mortality were 5.6 (95% CI 2.8 to 11.2) after adjusting for serum lactate and 5.3 (95% CI 2.5 to 10.9) after adjusting for mixed venous oxygen saturation. Error bars indicate 95% CI. Data from only one hospital were used for analyses of serum lactate and mixed venous oxygen saturation, resulting in wider 95% CIs.

We also examined the association of hospital mortality and renal failure with the six-hour postadmission physiological variables. As expected, the mortality was lower in patients with higher measured cardiac indices, mixed venous saturation, normal lactate values and higher calculated oxygen delivery at six hours. However, the mortality was significantly lower in patients unexposed to inotropes even in the presence of six-hour measured physiological variables lower than recommended threshold values. The odds of dying in the hospital were four to eight times higher in the inotrope exposed group than in the unexposed group for similar levels of measured physiologic variables (Figure 2). A similar relationship was found for renal dysfunction (Figure 3).

As a low preoperative left ventricular ejection fraction may have increased the likelihood that a patient would be treated with inotropes, the effect of inotropes on mortality in the 232 patients with preoperative left ventricular fractions < 30% was examined. In this subgroup, patients who received inotropes had significantly higher mortality than patients who did not, with an odds ratio OR of 14.7 (95% confidence interval (95% CI) 2.0 to 11.1).

After adjusting for differences in Parsonnet score, left ventricular ejection fraction, perioperative intraaortic balloon pump use, bypass time, reoperation and cardiac index, exposure to inotropes was associated with increased hospital mortality (adjusted OR 2.3, 95% CI 1.2 to 4.5; *P *= 0.01) (Table 3). Receipt of inotropes was also significantly associated with increased odds of renal dysfunction (adjusted OR 2.7, 95% CI 1.6 to 4.7; *P *< 0.001) (Table 4). There were no differences in these associations when treatment hospital, preoperative renal dysfunction or aprotinin use was included in the regression analysis. As these variables did not confound the association between the exposures and outcomes, they were not included in the final regression model.

**Table 3:** Multivariable analysis of association between inotrope exposure and hospital mortality^a^

Variable	Crude odds ratio	Adjusted odds ratio (95% CI)	*P *value
Inotrope status			
Inotrope unexposed (ref)	1.0	1.0	
Inotrope exposed	9.1	2.3 (1.2 to 4.5)	0.01
Parsonnet score			
0 to 9 (ref)	1.0	1.0	
10 to 14	2.4	1.8 (0.7 to 4.6)	0.24
15 to 19	3.1	1.8 (0.7 to 4.9)	0.23
20 to 29	8.3	4.6 (2.0 to 10.9)	< 0.001
≥ 30	24.6	11.2 (4.6 to 27.5)	< 0.001
Ejection fraction			
LVEF ≥ 30%	1.0		
LVEF < 30%	2.2	1.5 (0.9 to 2.7)	0.15
Intraaortic balloon pump use			
No perioperative IABP use	1.0		
Perioperative IABP use	8.2	3.3 (1.9 to 5.8)	< 0.001
Bypass time^b^	1.6	1.3 (1.1 to 1.4)	< 0.001
Reoperative status			
No reoperation	1.0		
Reoperation	6.1	4.7 (2.3 to 9.3)	< 0.001
Cardiac index at six hours, L/min			
Cardiac index ≥ 2.2 (ref)	1.0	1.0	
Cardiac index < 2.2	2.8	1.7 (0.93 to 1)	0.09

^a^LVEF, left ventricular ejection fraction; IABP, intraaortic balloon pump; ref, reference value; CI, confidence interval; ^b^modelled linearly as an increase in bypass time of 30 minutes.

**Table 4:** Multivariable analysis of association between inotrope exposure and renal dysfunction^a^

Variable	Crude odds ratio	Adjusted odds ratio (95% CI)	*P *value
Inotrope status			
Inotrope unexposed (ref)	1.0	1.0	
Inotrope exposed	7.5	2.7 (1.5 to 4.6)	< 0.001
Parsonnet score			
0 to 9 (ref)	1.0	1.0	
10 to 14	1.5	1.2 (0.7 to 2.4)	0.59
15 to 19	2.1	1.4 (0.7 to 2.9)	0.35
20 to 29	3.6	2.3 (1.2 to 4.3)	0.01
≥ 30	6.5	2.8 (1.3 to 6.1)	0.007
Ejection fraction			
LVEF ≥ 30%	1.0		
LVEF < 30%	1.6	1.0 (0.6 to 1.7)	0.97
Intraaortic balloon pump use			
No perioperative IABP use	1.0		
Perioperative IABP use	5.4	2.7 (1.6 to 4.7)	< 0.001
Bypass time^b^	1.5	1.2 (1.1 to 1.4)	< 0.001
Reoperative status			
No reoperation	1.0		
Reoperation	4.5	2.3 (1.2 to 4.5)	0.02
Renal dysfunction			
Normal preoperative renal function	1.0		
Preoperative renal dysfunction	3.7	1.7 (0.8 to 3.6)	0.14
Cardiac index at six hours, L/min			
Cardiac index ≥ 2.2 (ref)	1.0	1.0	
Cardiac index < 2.2	1.7	1.0 (0.6 to 1.9)	0.88

^a^LVEF, left ventricular ejection fraction; IABP, intraaortic balloon pump; ref, reference value; CI, confidence interval; ^b^modelled linearly as an increase in bypass time of 30 minutes.

Using greedy one-to-five matching, 123 inotrope-exposed patients were matched to 123 unexposed patients using one-digit matching only. Only preoperative left ventricular ejection fraction was statistically significantly different between the two groups, although equal numbers of patients had left ventricular ejection fractions < 30%. Hospital mortality, renal dysfunction, ICU and hospital length of stay were significantly worse in the patients exposed to inotropes (Table 5).

**Table 5:** Baseline characteristics and outcomes for propensity-matched groups^a^

Characteristics	No inotropes (*n *= 123)	Inotropes (*n *= 123)	*P *value
Demographics			
Mean age (± SD)	67.2 (11.5)	67.3 (10.9)	0.97
Age ≥ 80 years, *n *(%)	18 (14.6)	14 (11.4)	0.45
Females, *n *(%)	49 (39.8)	43 (5.0)	0.48
Medical history, *n *(%)			
Prior cardiac surgery	5 (4.1)	6 (4.9)	1.0
Hypertension	84 (68.3)	78 (63.4)	0.53
Diabetes	40 (32.5)	43 (35.0)	0.78
Atrial fibrillation	17 (13.8)	15 (12.2)	0.84
Preoperative hospitalisation for CHF	20 (16.3)	11 (8.9)	0.12
Preoperative renal dysfunction	9 (7.3)	10 (8.1)	1.0
Preoperative dialysis	1 (0)	0 (0.8)	0.32
LVEF < 30%	25 (20.3)	25 (20.3)	1.0
Mean LVEF (± SD)	50.6 (13.9)	45.8 (15.2)	0.02
Procedure-related variables			
Mean Parsonnet score (± SD)	14.1 (8.7)	14.4 (9.2)	0.78
CABG only, *n *(%)	82 (66.7)	85 (69.1)	0.80
Other procedure, *n *(%)	41 (33.3)	38 (30.9)	0.80
Perioperative IABP, *n *(%)	3 (2.4)	4 (3.2)	1.0
Emergency operation, *n *(%)	14 (11.4)	17 (13.8)	0.70
Reoperation, *n *(%)	4 (3.3)	9 (7.9)	0.23
Mean bypass time, minutes (± SD)	99.3 (28.7)	98.6 (32.9)	0.83
Severe postoperative bleeding, *n*(%)	2 (1.6)	6 (4.9)	0.29
Outcomes			
Died, *n *(%)	1 (0.8)	10 (8.1)	0.01
Renal dysfunction, *n *(%)	2 (1.6)	12 (9.8)	0.01
Median ICU length of stay (IQR)	1.0 (0.9,1.8)	1.8 (0.9,3.2)	< 0.0001
Median hospital length of stay (IQR)	8.0 (6.0,11.0)	9.0 (6.0,18.0)	0.03

^a^CHF, congestive heart failure; LVEF, left ventricular ejection fraction; CABG, coronary artery bypass graft; IABP, intraaortic balloon pump; SD, standard deviation; IQR, interquartile range.

## Discussion

In this observational study, postoperative inotrope exposure was associated with increased hospital mortality and renal dysfunction in cardiac surgery patients. Hospital mortality and renal dysfunction were consistently lower in patients unexposed to inotropes, even when their six-hour physiological variables were lower than the targets recommended in the literature [3,10]. The relationship between inotrope exposure and poor outcomes remained significant after adjusting for differences in Parsonnet score, left ventricular ejection fraction, perioperative intraaortic balloon pump use, bypass time, reoperation and cardiac index. In addition, an analysis using propensity score matching produced similar results.

The demonstration of poorer outcomes in patients exposed to inotropes is consistent with the results demonstrated in several previous studies. An observational study of patients who received dobutamine after cardiac surgery demonstrated increased cardiac morbidity after the data were adjusted for confounders [8]. Milrinone has also been described as being associated with an increased risk of postoperative atrial fibrillation in a cardiac surgery population [12]. Further evidence that inotropes may be harmful can be found in the heart failure literature. Randomised, controlled trials of patients with decompensated heart failure treated with phosphodiesterase inhibitors versus placebo revealed that those in the treatment group experienced more episodes of hypotension and cardiac arrhythmia and had higher mortality rates [13-15]. Furthermore, despite promising initial results, levosimendan, a new class of inotrope, was not shown to be superior to dobutamine in a randomised, controlled trial of patients with acute heart failure and left ventricular ejection fractions < 30% [16]. Researchers who conducted a systematic review of controlled trials of β-adrenergic agents compared to either placebo or an active agent in patients with heart failure concluded that there is very little evidence that treatment improves symptoms or patient outcomes and may in fact be harmful [9]. Inotrope use was also associated with increased mortality in a recent observational study of heart failure with an effect size similar in magnitude to that found in our study [17].

Since the 1970s, a number of randomised trials have been undertaken in medical and surgical patients to investigate whether targeting specific resuscitation goals, such as cardiac output and oxygen delivery, would improve patient outcomes [18]. Achieving the prespecified goals often involved the use of inotropic medications to increase cardiac output and oxygen delivery. Such therapy, referred to as 'goal-directed therapy', has been associated with improved outcomes, primarily in patients with sepsis and in certain high-risk surgical patient populations [10,19]. However, these trials differed with regard to patient mix, physiologic targets, therapies used and management of control arms [20].

Four controlled studies have been published in which a goal-directed therapy protocol was used in the cardiac surgery setting [21-23]. The trials differed with regard to targeted goals, therapeutic protocols and use of inotropic medications. Two of the trials, which employed mainly fluid infusions, demonstrated improved physiological goals with minimal catecholamine use. The largest trial, which targeted mixed venous oxygen saturation, demonstrated shortened hospital stay and less morbidity, but these outcomes were associated with increased catecholamine use. The last trial, which enrolled 30 patients in total, demonstrated no significant difference in outcomes but did require a more intense inotrope regimen to attain the specified goals. Despite demonstrating improved clinical outcomes, all four studies were underpowered to detect any difference in mortality. Furthermore, two of the four protocols required greater catecholamine doses to achieve their goals.

Several mechanisms may explain the increased mortality observed in patients exposed to inotropes. The two most common side effects of inotropic medications are increased myocardial oxygen consumption and cardiac arrhythmia. Both of these side effects may lead to poor cardiac performance [7]. Alternatively, low cardiac output may be due to mechanical obstruction, as in cardiac tamponade, which may require surgical intervention. The use of inotropes in these situations may transiently improve the haemodynamic state but ultimately lead to further harm, as appropriate diagnosis and treatment may be either delayed or missed altogether. Furthermore, catecholamine use has been associated with reduced metabolic efficiency by promoting fatty acid oxidation over that of glucose. This may be a further impediment to optimal cardiac performance. Catecholamine use has also been associated with bacterial growth, increased bacterial virulence, biofilm formation, insulin resistance and hyperglycaemia, all of which may contribute to poor outcomes [25].

Our study has several strengths. First, our results are less likely to be biased by a single centre's practice pattern, as the patients were recruited from two centres. Second, as we routinely collected postoperative physiological data, we were able to adjust for important haemodynamic variables. Finally, because extensive preoperative, intraoperative and postoperative data were collected, we were able to control for many potential confounding factors.

The study's main limitation is that it is an observational study, and thus the associations could be due to residual confounding. Specifically, we may not have fully adjusted the data for confounding by indication and confounding due to severity of illness. To minimise confounding, we performed multiple different analyses, including a propensity-matched analysis.

## Conclusions

The results of our study demonstrate that inotrope exposure was associated with increased hospital mortality and renal dysfunction in cardiac surgery patients. The observational nature of the data and the potential for confounding precludes any final conclusions about a causal relationship. Nevertheless, the significant practice variations reported in the literature, and the consistency of our results with those reported in the cardiac surgery and heart failure literature, demonstrate the need for future research [4-6,26]. As inotropes may be associated with increased morbidity and mortality, adequately powered, randomised, controlled trials are needed to clarify the risks and benefits of inotrope use in cardiac surgery patients.

## Key messages

• Postoperative inotrope exposure was independently associated with hospital mortality and renal dysfunction.

• The increased hospital mortality and renal dysfunction in patients exposed to inotropes are observed even when recommended six-hour physiological variables are achieved.

• Patients unexposed to inotropes with six-hour physiological variables below recommended targets had lower mortality than patients exposed to inotropes who achieved these targets.

## Abbreviations

CABG: coronary artery bypass and graft; CI: confidence interval; SD: standard deviation.

## Competing interests

The authors declare that they have no competing interests.

## Authors' contributions

JS and SD contributed to the study design and analysis as well as the drafting of the manuscript. JS, BD, CWT and AA contributed to the acquisition of data. All of the authors approved the final manuscript.
